# How Does Culture Shape Creativity? A Mini-Review

**DOI:** 10.3389/fpsyg.2019.01219

**Published:** 2019-05-28

**Authors:** Yong Shao, Chenchen Zhang, Jing Zhou, Ting Gu, Yuan Yuan

**Affiliations:** ^1^ College of Economics and Management, Guangxi Normal University, Guilin, China; ^2^ School of Art, Nanjing University, Nanjing, China; ^3^ Department of Art Design, Changzhou Art Vocational College of Jiangsu Province, Changzhou, China; ^4^ Department of Information Media, The City Vocational College of Jiangsu, Suzhou, China; ^5^ School of Rehabilitation Science, Jiangsu Provincial Key Laboratory of Special Children’s Impairment and Intervention, Nanjing Normal University of Special Education, Nanjing, China

**Keywords:** culture, creativity, conceptualization, thinking pattern, creative process

## Abstract

The purpose of this study was to examine how culture shapes creativity by reviewing empirical findings across diverse studies. The impact of culture on creativity is typically manifested in three ways: (1) people from different cultures or settings have distinct implicit and/or explicit conceptions of creativity; (2) individuals from different cultures, particularly those from individualist and collectivist cultures, show differences in preferred creative processes and creative processing modes (e.g., usefulness seems more important than novelty in the East, whereas novelty seems equally important as usefulness, if not more so, in the West) when they are engaged in creative endeavors; (3) creativity may be assessed using different measures based on culture-related contents or materials, and findings are accurate only when culturally appropriate or culturally fair measures are used. Potential implications and future directions are also proposed.

## Introduction

Creativity, a key engineer for facilitating social harmony, sustainable human development, technological invention and scientific revolution, is manifested in human activities at different levels, from everyday life to advanced technological industries. To date, there is no consensus-based definition of creativity; however, according to a standard definition, creativity is often perceived as the ability to produce something new/novel and appropriate/useful. At present, unprecedented significance is being attributed to creativity due to the implementation of policy-based, innovation-oriented national development strategies and increasingly pressing global issues, such as global warming and terrorism. Perhaps for these reasons, the study and application of creativity have received considerable attention in the past 10 years, and many critical findings regarding creativity have been revealed through a variety of research methods and approaches. Although these findings play an increasingly important role in understanding and developing creativity, they are largely isolated and fragmented because of the various approaches and methods used. Creativity, as a key product of human culture and a tool for enriching culture, has an extremely intimate but complex relationship with culture. To obtain a comprehensive understanding of the nature of creativity, this work attempted to integrate available studies from a cultural and cross-cultural research perspective. In a sense, creativity is inherent to culture. Simply speaking, if culture is the “background,” then creativity is the “object” that is likely to become a new “background” for emerging and forthcoming “creativity (objects).” Nobody can live well and be creative without the involvement of culture. Accordingly, the present study attempted to identify how culture molds creativity from a (cross-)cultural psychology perspective.

Creativity is deeply rooted in all cultures, but its definition and attributes vary across cultures. According to the literature, the dichotomy of “the West” and “the East” is one of the most influential approaches in characterizing (potential cultural) differences in understanding and defining creativity. “The East” commonly refers to Asian countries, especially East Asian countries such as China and other countries influenced by its culture, such as Japan or Korea; they possess general similarities in social and cultural aspects that differ from those of “Western” countries. The mentioned countries are often considered to largely represent “collectivist cultures” (i.e., cultures that emphasize that collective interests should override individuals’ interests and that fitting in with the collective is more important than being unique) and share a similar tradition that traces its origin from Asian thought, such as Taoism, Buddhism, and Confucianism. In contrast, “the West,” although usually considered to reflect “individualist cultures” (i.e., those that value the individual’s goals and interests over the group’s collective interests and goals; Xie and Paik, 2019), usually refers to the US, Western Europe, Canada, Australia, and New Zealand, which are closely linked to ancient Greece and the ideas of Christianity, Judaism, and rationality (Weiner, 2000; see Dubina and Ramos, 2016). The plausibility of such clustering of the East and the West has been substantially supported by several large-scale studies, such as the World Value Survey (Inglehart et al., 1998) and the GLOBE project survey (House et al., 2004; see Xie and Paik, 2019).

In a review of previous studies, we found that some pioneering studies have probed the link between culture and creativity from various perspectives. These studies included two fascinating approaches: a comparison of cross-cultural creativity between aboriginals from different cultural backgrounds and the investigation of the effects of multicultural experiences, such as studying abroad, and cultural priming on creativity. Leung and Chiu (2010), for example, asked European American undergraduates to complete a creative writing task (followed 5–7 days later by a creative analogies construction task) immediately after exposure to visual materials that mirrored either American or Chinese cultures or a hybrid culture created by the fusion of American and Chinese cultures. The researchers not only empirically documented a robust facilitative role of multicultural experience in both immediate and delayed creative performance but also found that this effect was primarily mediated by the generation of uncommon and unconventional ideas and by enhancing receptiveness to ideas that are natively rooted in foreign cultures and modulated by individuals’ need for cognitive closure and existential terror. Similarly, through five studies using a multimethod approach, Maddux and Galinsky (2009) empirically revealed the positive association between time spent living abroad (not traveling abroad) and creativity, demonstrating that foreign living experiences and the experience of adapting to a foreign culture temporarily boosted creativity in individuals who had lived abroad. Although considerable studies of this kind have reliably replicated the effects of exposure to foreign culture on facilitating creativity, they require integration and a more comprehensive perspective. Two recent theoretical studies (Shen and Yuan, 2015; Huang et al., 2018) attempted to integrate these “isolated findings”; however, they mainly focused on the influence of different aspects of culture (i.e., cultural values/norms and activities, multicultural learning, or cultural artifacts/products) or multicultural experiences on creativity (as an entity), without evaluating the effect of culture on different aspects of creativity. Nonetheless, creativity as a multifaceted and complex construct naturally involves distinct representation across different levels, from conceptual analysis to experimental manipulation and practical assessment. In fact, an increasing number of studies have been criticized for utilizing a “Western” (or American) framework to conceptualize and measure creativity (Glăveanu, 2010). Therefore, to deepen the understanding of the relationship between culture and creativity, particularly the role of culture in modifying and nurturing creativity, the major roles of culture in conceptualizing, manipulating, and measuring creativity were carefully identified.

The remainder of this research is organized as follows. Section “Culture Underwrites the Definitions of Creativity” mainly illustrates the role of culture in conceptualizing or defining creativity. Next, we present recent findings on the influence of culture on the creative process, followed by the effect of culture on assessing creativity or developing creativity measures. Finally, the study concludes with some proposed directions for future studies.

## Culture Underwrites the Definitions of Creativity

Creativity is bound to culture. To systemically or scientifically investigate a new construct, the first order of business is to establish a definition of the construct. Without exception, a key to scientifically demystifying the construct of creativity is to conceptualize or define creativity. Although defining creativity is easy, establishing a consensual definition of creativity is not. Previous studies have acknowledged that a collection of creativity conceptions proposed by earlier psychologists has been compiled into a book (see Treffinger, 1996), indicating that there have been considerable attempts and efforts to explicitly define creativity. For example, working from the perspective of intersubjectivity, Glăveanu (2010, p. 157) conceived of creativity as a complex phenomenon leading to “the generation of new and valuable artifacts by working with ‘culturally impregnated’ materials within an intersubjective space.” However, the standard definition of creativity argues that creativity requires both originality (also called novelty, newness, or uniqueness) and effectiveness (also called utility, usefulness, appropriateness, value, or meaningfulness) (Runco and Jaeger, 2012, p. 92). In contrast to considering creativity as an intrapersonal cognitive process or performance or as an individual’s personality or abilities (Williams and Yang, 1999, p. 378), creativity is assumed to be a complex, multivariate construct or phenomenon that refers to the “interplay between ability and process by which an individual or group produces a perceptible outcome or product that is both novel and useful as defined within some social context” (Plucker et al., 2004, p. 90). Perhaps because so many different definitions of creativity are available and because of the lack of universal agreement regarding such definitions, individuals across cultures conceptualize creativity differently and sometimes use context- or culture-specific theories of creativity as general theories or definitions (Lee et al., 2015). A review of the existing literature found that some definitions of creativity concerned the nature of dynamic thought processes and the intellectual capability used to produce insights or creative solutions to problems; some primarily focused on the personal characteristics (personality or traits) and cognitive abilities of individuals; and still others targeted the products or outcomes of creative attempts (Martins and Terblanche, 2003). Importantly, some variability is found in explicit conceptions of creativity across cultures or countries. Typically, these suggest that Western cultures attach more importance to process- and product-based creativity and highlight the pragmatic, problem-solving outcome of creativity and that Eastern cultures have great interest in creative spirits and person-based creativity, treating creativity as a form of revelation or rediscovery (Westwood and Low, 2003) and emphasizing the role of creativity in facilitating personal fulfillment and enlightenment or the self-expression of an inner essence or ultimate reality (Lubart, 1999; Glăveanu, 2010).

Numerous conceptual constructs in psychology can be studied and depicted as explicit (based on domain experts and/or theories) or implicit (derived from laypersons’ or individuals’ belief systems). Creativity is no exception. In contrast to explicit theories, which rely heavily on experts’ data-driven theories regarding creativity, implicit theories of creativity preexist in people’s minds and only need to be discovered (Shen et al., 2018b). Implicit theories are regarded as having great practical and theoretical importance for formulating common cultural views on creativity and understanding how individuals perceive their own beliefs regarding creativity (Rudowicz, 2003; Shen et al., 2018b). Consistent with the numerous studies that have attempted to determine the explicit concepts of creativity, substantial evidence has demonstrated that implicit conceptions of creativity show some variability across cultures. According to Rudowicz (2003), the majority of studies concerning implicit theories of creativity have either focused on creative individuals (the traits or personality characteristics that typify a creative individual) or the conceptualization of creativity (what laypeople perceive creativity to be). For example, Sternberg (1985) reported that the implicit conception of creativity overlaps with but also distinctively differs from the conceptions of intelligence and wisdom. In Western studies, descriptions such as “curious,” “imaginative,” “independent,” “inventive,” “original,” “wide interests,” “nonconformist,” “individualistic,” “confident,” “assertive,” “daring,” “artistic,” “open-minded,” “intelligent,” “capable,” and “sense of humor” were frequently named as implicit personality characteristics that describe a creative individual (Runco and Bahleda, 1987; Rudowicz, 2003; Runco, 2014; Luescher et al., 2019). Using Chinese undergraduates as participants, Rudowicz and Yue (2000) demonstrated that Chinese college students from Beijing, Guangzhou, Taipei, and Hong Kong all named “originality,” “innovativeness,” “thinking,” “observational skills,” “flexibility,” “willingness to try,” “self-confidence,” and “imagination” as core characteristics of a creative person, with some regional differences (except in the Taipei sample) that attributed “wisdom,” “assertiveness,” and “individualism” to creativity. Additionally, Rudowicz and Yue (2002) surveyed 489 Chinese students and required them to name the most creative Chinese people in history and in modern times. Their results showed that Chinese youths’ perceptions of creative individuals focused more on an individual’s social influence or their potential or realized contributions to society through their creativity. Furthermore, Rudowicz (2003) noted that characteristics related to “artistic” and “sense of humor” were missing or almost nonexistent in Chinese perceptions of creativity and that “inspires people,” “makes contributions to society,” and “is appreciated by others” were uniquely Chinese views of creativity that were not reported among Westerners’ implicit conceptions of creativity. Overall, to Westerners, creativity implies a break with tradition and a move beyond what exists, whereas to Easterners, creativity suggests the reinterpretation or rediscovery of tradition. Relatedly, in the West, creativity is valued primarily for solving particular problems through insight or achieving personal success, whereas in the East, the value of creativity primarily lies in the social and moral contributions an individual can make to society (Rudowicz and Yue, 2000; Niu and Sternberg, 2006).

Taken together, although mounting studies have investigated the potential influence of culture or multiculturism on creativity (Leung et al., 2008; Leung and Chiu, 2010; Shen and Yuan, 2015; Chua, 2018), including conceptualizing creativity and showing the rich connotations and cultural variability of creativity, the majority of these studies focused on experts’ data-driven definitions of creativity and laypersons’ perceptions of creativity to identify explicit and implicit conceptions of creativity. Western notions of creativity primarily focus on creative processes and products at the explicit level and on achieving personal success and solving difficult problems at the implicit level, whereas the Eastern world strongly emphasizes the spirit of creativity and personal characteristics, either traits or abilities, at the explicit level and individuals’ moral and social contributions to society at the implicit level.

## Culture Underwrites Creative Processes

As a complex and multistage process, creativity is not integral to an entire process and may involve various subcomponents or subprocesses. In other words, creativity is a consequence of human thought that involves a variety of creative processes and operates on a set of existing representations, concepts, objects, symbols, rules, or notions. As mentioned above, creativity does not seem to appear in a vacuum or to be isolated from various materials. The materials that are involved in or processed during creativity include representations, concepts, objects, symbols, rules, or notions, which are actually derived from individual and group context-related experiences or cultures and undoubtedly involve elements that are more or less cultural. Nevertheless, the cultural effect of the materials processed during different stages or processes of creativity is not the point of interest in this study; rather, the point of special interest is the effect of culture on creative processes or stages across diverse contexts or cultures. However, this approach does not suggest that the processes underlying creativity in different cultures are completely distinct, without any standard or normative processes or stages; rather, it means that researchers from different cultures are inclined to study different creative processes or processing modes and assign different degrees of importance to the same aspect of the creative process.

Creativity embraces both novelty and usefulness. These concepts correspond to two critical processes of creativity: the generative process of acquiring and accessing information and knowledge and recombining them to produce new ideas and the exploratory process of searching one’s knowledge for novel and potentially useful combinations of ideas and judging the viability of potential solutions (Chua et al., 2015). Substantial evidence indicates that in Western or individualistic cultures (Xie and Paik, 2019, p. 7), greater importance is assigned to the novelty processing mode underlying creativity and to flexible, inferential processing, which is beneficial to generating more novel solutions; in contrast, Eastern or collectivist cultures attribute more significance to the processing mode of appropriateness or usefulness underlying creativity and value cautious, persistent processing, which is conductive to more useful solutions (Nijstad et al., 2010; Adair and Xiong, 2018). In fact, the emphasis on novel or “groundbreaking” outcomes fits better with the Western or individualist belief system, which is based on the ideals of individuality, freedom, and democracy. In contrast, the focus on usefulness reflects a strong reliance on tradition, and Eastern or collectivist societies, which are firmly grounded in the ideals of interdependence, cooperation, collectivity, and authoritarianism, have evolved a distinct perspective on the inherent meaning of uniqueness, originality, and/or novelty (Rudowicz, 2003; Kaufman and Lan, 2012).

Similarly, different degrees of significance have been attributed to radical creativity or innovation and incremental creativity or innovation. A gradual or incremental pattern dominates creativity in the East, while a pattern of radical creativity is the dominant pattern of creativity in the West (Shen et al., 2018b). Specifically, some studies have shown that in East Asian cultures, particularly in Chinese culture, there is both a strong desire for creativity and great fear and rejection of radical creativity (Paletz and Peng, 2008; Shen et al., 2018b). In two recent studies (Jarman, 2014; Shen et al., 2018b) that examined the psychological processes underlying creative insight, particularly insight experiences, researchers observed that small radical insights or radical restructuring (which, according to the Chinese, only occurs when solving brainteasers; see Luo and Knoblich, 2007) is different from the robustness of more radical forms of creativity (e.g., radical insight, restructuring or creativity) valued by Westerners (Jarman, 2014). Essentially, the cultural difference in preferred creativity processing patterns or creative processes is rooted in belief system differences between the East and the West. Specifically, in Eastern areas, creativity is characterized as an ongoing process involving “a circular movement in the sense of successive reconfiguration of an initial totality”; in contrast, in the West, creativity is considered “a linear movement towards a new point” and “an insightful production achieved by individuals engaged in a working process with a finite beginning and end” (Lubart, 1999, p. 341). There is a unique dialecticism called Chinese naïve dialecticism that is mainly derived from East Asian philosophical and religious traditions, such as Confucianism, Taoism, and Buddhism; as such, it deals with apparent contradictions by retaining the fundamental elements of opposing perspectives as a state of tension or conflict in which contradictions do not necessarily have to be resolved and opposites can coexist (Paletz and Peng, 2009; Shen et al., 2018b). Under such belief systems, almost all things that Westerners believe are radical are regarded by Chinese as incremental.

The four-stage approach of Wallas (1926), derived from Helmholtz’s ideas on the thought process involved in creative ideas (Rhodes, 1961; Sadler-Smith, 2015), is a heuristic working model that illustrates key cognitive processes of creativity; it states that the creative process can be divided into the stages of preparation, incubation, illumination, and verification (Shen et al., 2018a). Although Wallas’ four-stage model of creativity, the most widely cited framework for creativity in the West, has been used to describe the key processes underlying creativity in some contemporary Eastern studies on creative cognition, such as creative thinking and insight, cultural variation in the four stages of the creative process also exists; for example, relatively, the Western process model follows a cognitive problem-solving approach (a product-oriented definition of creativity; e.g., Dubina and Ramos, 2016), whereas the Eastern creative process highlights the emotional, personal, and intrapsychic aspects of creativity. Lubart (1999) cited two alternative models, contrasting Eastern processes of creativity with the Western four-stage model of creativity which were introduced in Maduro (1976) and Chu (1970). The four-stage model based on the Yoga Sutras (from Maduro, 1976) emphasizes self-will and the ceaseless effort, internal identification, personal insights, and social communication of personal realizations; these aspects are individually considered similar to the four stages of Wallas’ creative model (preparation, incubation, illumination, and verification) (Shen et al., 2018a). Different from the Western depiction of the creative process, mediation or mindfulness is also regarded as a key facilitator and sometimes as integral to the Eastern process of creativity. In addition, from a three-stage perspective, Chiu and Kwan (2010) assessed how culture impacts the creative process of authoring, editing, and accepting and revealed that existing knowledge of the established norms of one culture and cultural elements are not only reference points for determining the originality or uniqueness of new ideas but also serve as important sources of inspiration. Specifically, the cultural norms and elements in which an individual is located build perceptual and conceptual sets that may also result in mental impasses and impede the fluency of the problem-solving process. In contrast, exposure to a foreign culture could help expand the conceptual boundaries established in the individual’s culture, providing inspiration to break free from his/her culture’s limiting sets and initiating the creative reappropriation/synthesis of diverse ideas. One powerful avenue for creativity is the combination of disparate ideas from as diverse categories (a culture can be considered a category) as possible. Individuals exposed to different cultures have a great likelihood of generating novel or new things through combining different elements from the experienced cultures, which would be perceived as novel by individuals who have experience with only one or some of those cultures. Culture is believed to indirectly facilitate or impair the fluency of the creative process of idea authoring (i.e., authoring creative ideas) through moderating variables or intervening factors, such as the process of selecting, editing, and marketing new ideas (i.e., how ideas are edited and marketed) and the process of accepting or tolerating creative or novel ideas (e.g., the acceptability of novel ideas).

In summary, the role of culture in underwriting creative processes is primarily that culture both provides the fundamental materials and inputs that are processed in creativity and modifies the specific processes of creativity directly and/or indirectly. Cultural knowledge, such as awareness of cultural norms, simplifies, facilitates, or modifies some processes of creativity and provides reference points for these processes. In addition, culture may lead to different preferences in the selection and application of models of creative processes among different cultures and to different attributions of importance to the same creative process across cultures.

## Culture Underwrites the Assessment of Creativity

Through the use of creativity measures and assessment tools, creativity can be studied qualitatively and/or quantitatively. Most previous studies have shown that Westerners scored higher than their Eastern counterparts on various creativity measures or tests (for details, see the Singaporean bestseller *Why Asians Are Less Creative than Westerners*; Ng, 2001), resulting in an enduring controversy regarding whether Eastern or Asian populations lack creativity or are inferior in terms of creativity. However, an increasing number of studies demonstrate that although Western students perform better on many measures of divergent thinking and creative performance, this superiority is not consistent across all types of creativity measures (e.g., convergent thinking measures) and does not always appear for all dimensions of creativity assessment (e.g., originality, fluency, and flexibility; see Xie and Paik, 2019). For instance, Saad et al. (2015) reported that more ideas (assessed by two divergent thinking problems) were generated by Canadians (representing individualist culture), while Taiwanese (representing a collectivist culture) showed greater originality in their generated ideas. In a review based on 29 published papers, Xie and Paik (2019) reported mixed findings regarding the effect of individualist vs. collectivist cultures (West vs. East) on creativity, as assessed with a variety of creativity measures.

Given the complexity of conceptualizing creativity and the variation in creative processes across contexts and cultures, it is difficult to believe that assessing creativity is a simple thing. This difficulty in assessing creativity mirrors the diversity of cultures or contexts in which creativity is rooted and is reflected in the variation in creativity measures (for instance, see Cropley, 2000) and approaches to assessing creativity. As early as 1989, Torrance and Goff (1989), in an increasingly substantial body of evidence, listed more than 255 creativity assessment tools; the current number of creativity measures may be substantially higher. Overall, the four P’s of creativity – person, product, process and press – seems to be a more profitable framework for measuring creativity. Of the instruments developed to assess creativity, some attempt to evaluate the cognitive processes involved in creativity, such as creative insight; some are concerned with product-based creativity or creative performance/achievements; some focus on personal characteristics or personality traits; and others examine the impact of the environment (Feldhusen and Goh, 1995).

Culture is a key factor that influences cross-cultural assessment and determines individuals’ or groups’ creativity in diverse settings. In contrast with previous assertions that Western populations have higher scores on creativity tests compared with Asian or Chinese populations (Lubart, 1999; Kharkhurin and Samadpour Motalleebi, 2008), recent studies have revealed that individuals from tight cultures, as opposed to those from loose cultures, feel more confident and experience greater creative self-efficiency when they attempt creative tasks or measures within their own cultures (see Chua et al., 2015). More importantly, an increasingly substantial body of evidence reliably shows that Asian and Chinese populations exhibit greater creativity on some creative performance or thinking measures (e.g., Zhou et al., 1995; Xie and Paik, 2019). For example, by presenting six creative problems to Chinese and American college students, Chen et al. (2004) examined the potential influence of the folk tales (cultural compositions) that the participants had heard during their childhood on creative or insight-based problem-solving. They observed that more than 70% of Chinese undergraduate students were able to solve the Statue Problem, which is procedurally similar to the folk tales they heard during childhood; in contrast, fewer than 10% of their American counterparts solved this problem because they had not heard the relevant folk tales. The opposite performance pattern was observed for solving the Cave Problem, which was similar to stories the American students had heard during childhood but bore no similarity to any tale the Chinese students had heard before. This effect might be considered a familiarity effect (cultural customs) rather than truly superior creativity. However, another longitudinal study by Zhou et al. (1995) provides stronger evidence; in that study, a three-wave (longitudinal) China-German comparison of children’s creative performance (assessed individually using figural divergent thinking test, verbal alternative uses test, and technical problem-solving task) was conducted, and better figural creativity performance was found for Chinese elementary students than for their German counterparts (irrespective of grades), while the opposite trend was found for verbal convergent thinking performance. After reviewing 29 papers, Xie and Paik (2019) found no consistent findings regarding the relative strength of Westerners in creativity and innovation.

Findings of this type suggest that culturally appropriate measures are important for accurately assessing creativity and that culture may influence the precise assessment of creativity. The same issue also appears in the Torrance Tests of Creative Thinking (TTCT). For example, Lubart (1999) noted that the images and objects that are used to construct test questions or brief paper-and-pencil creativity tasks such as those on the TTCT may be culturally bound (e.g., Jellen and Urban, 1989; Rudowicz et al., 1995). Perhaps for these reasons, the seven-piece puzzle (tangram; e.g., Domino, 1980; Siew and Chong, 2014) and the Chinese ring puzzle (puzzle ring; see Hamel and Elshout, 2000) were developed by the Chinese to measure creativity and/or intelligence; in contrast, tasks involving Roman numerals (see Knoblich et al., 2001) and English letters (e.g., anagrams; see Bowden, 1997) were developed by Westerners to assess creativity in the West. Nevertheless, some issues persist due to the use of language or verbal forms to assess creativity across different cultures. A growing number of studies show that language learning can influence individuals’ creativity and that language, as an integral aspect of culture, may influence the generation of creativity (expressed in verbal forms). In a study of Hong Kong children that used the Chinese version of the TTCT as a creativity measurement tool, Rudowicz et al. (1995) found that “stimuli, in the form of pictures, presented in the verbal forms seem to relate to stories that are more familiar to American and European children than to Asian children” (p. 424; see also Lubart, 1999). In addition, most creativity measures are developed in the West, especially in the United States; thus, Westerners may be more familiar with them than Easterners are, which would account for the better performance among Westerners. Simply speaking, familiarity with the materials, especially verbal expressions or symbols, that are used to construct test questions and culture-related misinterpretations of the task (which result from the existence of different norms or conventions) will both influence the accuracy of creativity assessments.

In addition to objective measures of creativity, many techniques are relative (sample- or population-based) measures of creativity, including divergent thinking tests and consensual assessment techniques. As an example, in the evaluation of originality or uniqueness in divergent thinking tests, such as the consensual assessment method, cultural differences in widespread knowledge and social norms mean that a response that is considered highly original or unique in one culture will not necessarily be considered highly unique in other cultures or contexts. As mentioned in Jones and Shea (1974), “statistical frequency-based measures of originality that lump together the responses of different cultural groups tend to inflate the originality score of responses common in one group and to diffuse culture-specific originality” (Lubart, 1999, p. 347). Additionally, the cultural background of the raters influences their ratings, a finding that has been better illustrated for differences in creativity or originality according to ethnicity (e.g., Kaufman et al., 2004; Kaufman, 2006) and gender (Gralewski and Karwowski, 2013), in which individuals of different genders or ethnicities could be considered members of different (sub)cultures.

The influence of culture on the assessment of creativity is typically manifested in two points below. On the one hand, culture exerts great impacts on the construction of creativity measurement instruments due to the cultural characteristics of the materials constructed as test questions or items. On the other hand, culture influences the expression or output of individuals’ creative ideas due to cultural familiarity or performance bias resulting from the application of culturally inappropriate creativity measures. Additionally, culture plays important roles in the subjective or relative ratings of subjective creativity and in some dimensions of creativity tests, especially population- or sample-based originality ratings.

## Concluding Remarks

Creativity is complex and culture-sensitive. In this review, cross-cultural differences in conceptualizing, processing, and measuring creativity were discussed and summarized by contrasting Eastern and Western cultures. Our research provides substantial evidence supporting the profound role of culture in defining and assessing creativity and underwriting creative processes. Consistent with our finding, Lubart (1999) argued that culture might influence creativity, and this influence might manifest as people from different contexts or cultures having distinct implicit and/or explicit concepts of creativity and people from various contexts or cultures adopting or preferring to use different psychological processes, such as tingeing, in their creative endeavors. Because the definition and assessment of creativity are highly dependent on culture, most of the observed differences in actual creativity could be the result of cultural differences. Future studies should take greater efforts to carefully examine the influence of culture on the “consensus components or operations” of creativity or the standard processes or criteria of creativity. In addition to the fact that culture, including language and environment, may nurture creativity, culture may also exert a robust role in the assessment of creativity. Future studies could empirically investigate the roles of culture, including but not limited to multicultural experiences and language learning, that have emerged in the past 10 years in underwriting the conceptualization, processing, and assessment of creativity. Additionally, other studies could take greater efforts to demystify the cross-cultural neural underpinning of creativity using high-density brain potentials or neuroimaging measures, given that two recent studies have started to explore the neural correlates of cross-cultural differences in creativity (Ivancovsky et al., 2018, 2019).

## Author Contributions

All authors listed have made a substantial, direct and intellectual contribution to the work, and approved it for publication.

### Conflict of Interest Statement

The authors declare that the research was conducted in the absence of any commercial or financial relationships that could be construed as a potential conflict of interest.
